# An Estimation of Mortality Risks among People Living with HIV in Karnataka State, India: Learnings from an Intensive HIV/AIDS Care and Support Programme

**DOI:** 10.1371/journal.pone.0156611

**Published:** 2016-06-02

**Authors:** Prakash Javalkar, Ravi Prakash, Shajy Isac, Reynold Washington, Shiva S. Halli

**Affiliations:** 1 Karnataka Health Promotion Trust, Bangalore, India; 2 University of Manitoba, Winnipeg, Canada; Iranian Institute for Health Sciences Research, ACECR, ISLAMIC REPUBLIC OF IRAN

## Abstract

**Background:**

In Indian context, limited attempts have been made to estimate the mortality risks among people living with HIV (PLHIV). We estimated the rates of mortality among PLHIV covered under an integrated HIV-prevention cum care and support programme implemented in Karnataka state, India, and attempted to identify the key programme components associated with the higher likelihood of their survival.

**Methods:**

Retrospective programme data of 55,801 PLHIV registered with the Samastha programme implemented in Karnataka state during 2006–11 was used. Kaplan-Meier survival methods were used to estimate the ten years expected survival probabilities and Cox-proportional hazard model was used to examine the factors associated with risk of mortality among PLHIV. We also calculated mortality rates (per 1000 person-year) across selected demographic and clinical parameters.

**Results:**

Of the total PLHIV registered with the programme, about nine percent died within the 5-years of programme period with an overall death rate of 38 per 1000 person-years. The mortality rate was higher among males, aged 18 and above, among illiterates, and those residing in rural areas. While the presence of co-infections such as Tuberculosis leads to higher mortality rate, adherence to ART was significantly associated with reduction in overall death rate. Cox proportional hazard model revealed that increase in CD4 cell counts and exposure to intensive care and support programme for at least two years can bring significant reduction in risk of death among PLHIV [(hazard ratio: 0.234; CI: 0.211–0.260) & (hazard ratio: 0.062; CI: 0.054–0.071), respectively] even after adjusting the effect of other socio-demographic, economic and health related confounders.

**Conclusion:**

Study confirms that while residing in rural areas and presence of co-infection significantly increases the mortality risk among PLHIV, adherence to ART and improvement in CD4 counts led to significant reduction in their mortality risk. Longer exposure to the intervention contributed significantly to reduce mortality among PLHIV.

## Introduction

Globally, it is estimated that 35.3 million [32.2 million–38.8 million] people were living with HIV (PLHIV) by the year 2012.[1] At the same time while about 1.5 million people die from HIV/AIDS-related causes, about 2.1 million new cases of HIV were detected around the world.[1] Although, the burden of the epidemic varies considerably between countries and regions; the HIV prevalence in India was estimated to be 0.27 percent by 2012.[2] With an estimated 2.1 million people living with HIV and AIDS, India is the country with third highest estimated number of PLHIV after South Africa and Nigeria.[3]

Despite having a high level of adult HIV prevalence, India has witnessed a gradual decline in the adult HIV prevalence from an estimated level of 0.41 percent in 2001 to 0.36 percent in 2006, and 0.27 percent in 2011 and thereafter.[4] Due to continued efforts in implementing the focused HIV/AIDS prevention intervention, India could manage to demonstrate the declining trends of estimated number of PLHIV (from 2.3 million in 2006 to 2.1 million in 2011). However, four high prevalence states of South India (Andhra Pradesh, Karnataka, Tamil Nadu, and Maharashtra) still account for more than half of the total HIV-infected population (56 percent) in the country.[5] Using the WHO/UNAIDS Workbook method, the National AIDS Control Organization (NACO) ranks Karnataka state fifth in India in terms of HIV prevalence (0.53 percent) by the year 2011.[2] Barring the three high prevalence states located in the north-east part of the country, Karnataka stands in second place, immediately after Andhra Pradesh (0.59) in terms of HIV-prevalence.[2] [4]

Although, the HIV prevalence among the adult population was considerably higher in the southern states of India, including Karnataka, the recent estimates give an indication of a reduction in the epidemic in the high prevalence states. For instance, India has demonstrated an overall decrease of 57% in estimated annual new HIV infections (among adult population) during the last decade from 2.74 lakhs in 2000 to 1.16 lakhs in 2011.[6] The significant contribution to this decline comes from the high prevalence states where a reduction of 76 percent noticed during the same period.[4] A major reason for the success has been the sustained commitment of implementing an effective HIV/AIDS prevention and care and support interventions among key populations having the components of regular outreach and clinic visits, condom distribution, HIV testing, linkages to care and support programme and reduction in loss to follow-up which latter acted as gateways for HIV into the general population.[7] This is one of the most significant evidence of the impact of the various interventions and scale-up strategies under the national prevention programme implemented through NACO.

Along with the other international donors, the United States Aid for International Development (USAID), as part of the President’s Emergency Plan for AIDS Relief (PEPFAR), also supported the Government of India’s efforts to reduce HIV/AIDS prevalence and mitigate the impact of the disease, especially in the four south Indian states. *Samastha*, the USAID/India funded the five-year HIV prevention, care and support project was one of the such projects implemented in Karnataka and coastal Andhra Pradesh (AP) aimed to reduce transmission and mitigate the impact of HIV in selected 15 high prevalence districts in Karnataka and four coastal districts in AP with a focus on rural, most at-risk and under-served populations.[8] Implemented by the Karnataka Health Promotion Trust (KHPT), Bangalore, India, the intervention in Karnataka mostly focused on implementation of comprehensive HIV-prevention and care support programme in the selected rural areas of the districts. The same in AP only dealt with the care and support component.

The project interventions included ‘link worker’ outreach covering about 2300 villages[9]; integrated positive-prevention and care drop-in centres to provide outreach services including counseling, linkages to treatment, nutrition and social schemes, and family level HIV testing for people living with HIV; and health system strengthening to scale up and maintain quality at the Government anti-retroviral treatment centres (ART), intensify the TB-HIV cross-referrals, enhance quality of community care centres and integrate the prevention of parent to child transmission programme with the national rural health mission.[8]

The purpose of this study is to estimate the risk of mortality among the PLHIV registered with the programme. This study also intends to present the adjusted survival probability of PLHIV in the form of expected number of surviving person per 1000 person-years in the presence of different intermediate factors that affect the likelihood of survival of PLHIV. These factors included linkages to ART, the CD4 count levels, the presence of other diseases like tuberculosis (TB), and duration of exposure to the intervention. Till date, only a few studies have made such systematic attempt to measure mortality rate using the programme monitoring data depicting the linkages between programme exposure components and HIV/AIDS-related deaths in India.[10–12] The findings of this study might prove useful for national HIV programme (prevention-care treatment-social support) to focus on the main aspects of care and support that has a larger contribution to the healthy survival of PLHIV.

## Data and Methods

### Ethical statement

For the present study we used secondary data obtained from computer management information system (CMIS) of Samastha project. CMIS data has been conceived as an important source for programme monitoring and improving the outcomes. The registration into the CMIS was completed only after initial rapport building and with written consent. According to the most recent peer reviewed publication using the same data, as used in our analysis, it is confirmed that the project conforms to the ethical standards set under the Helsinki Declaration.[9] Moreover, an approval was previously sought from the Institutional Review Board of St. John Medical College, Bangalore, to use the data for an already published research study.[13] Therefore, for our study, no separate approval was obtained. In the analysis, no personal identifiers were included to maintain the data confidentiality.

### Data description

In total, 55801 PLHIV were registered in the programme during its five-year implementation during October 2006 to September 2011 and were included in the study. The web-based system allowed the project to monitor the services offered in different facilities as well as during outreach by assigning a Unique Identity Numbers (UID) to every individual. Demographic characteristics, HIV testing, clinical and treatment details and other psychological and social information for PLHIV were collected during the initial registration at the centers (CCC and IPPCC) by the team of doctors or nurses, counselors and outreach workers using the standard format. Subsequently, all other medical and non-medical services provided periodically to the PLHIV were captured using separate visit forms along with the follow-up of referral to HIV-related services like linkages with ART centers, CD4 facilities, and TB diagnosis/treatment. A separate register was maintained to record details of the deaths among registered PLHIV. Data collected using paper-based registers/formats were updated continuously at the facilities by data entry operators under the supervision of the monitoring and evaluation officer. Data synchronization at the server was done on a weekly basis. A detailed description of the project can be found in the project evaluation report of the USAID.[8]

### Measures

The mortality (death) rate among the PLHIV was the primary outcome of interest. However, the analysis included a few socio-demographic, clinical and programme exposure related characteristics that affect the likelihood of survival among PLHIV. For instance, while age (<18 years; 18 years and above), sex (male; female), marital status at the time of registration (never married; ever married), educational status (non-literate; literate), employment status (not working/unemployed; working/employed), place of residence (rural; urban) were the socio-demographic characteristics of PLHIV included in the analysis; the clinical characteristics included ART status (not on ART; on ART), CD4 count at diagnosis and prior to death (not done; <100; 100–249; 250–349; and 350+), and symptoms of TB (yes; no). The two programme characteristics, namely, duration of exposure to the programme (<6 months; 6–11 months; 12–24 months; 24+ months) and duration since diagnosed with HIV (continuous variable) were also included in the analysis.

### Analysis

Bivariate and multivariate analyses were performed. The analysis presented in the paper demonstrated the differential in the characteristics of registered PLHIV, who died and survived (alive) until the end of the project period, on selected socio-demographic, clinical and programme exposure related characteristics. Z-test was performed to compare the profile of individuals across the two groups. The Kaplan-Meier (KM) method was used to estimate the survival probabilities. These probabilities were further stratified across different population sub-groups classified by their sex, ART status, CD4 count, and programme exposure and were adjusted for the effect of their place of residence (rural/urban). There results are presented in the form of survival curves. Adjusted cox proportional hazard model was used to examine the factors associated with risk of mortality. The results were adjusted for the selected socio-demographic, clinical and programme exposure characteristics mentioned above. Results are presented in the form of hazard ratio (HR) and confidence intervals (CI). The survival time was measured as the duration between first time tested HIV positive to the reported date of death for PLHIV, who died, or the date of the last recorded date of contact who were censored (either alive till the end of the study period or lost to follow-up). All statistical analyses were performed using STATA software (version 14.0).[14]

## Results

A total of 55,801 PLHIV registered with the programme were included in the analysis, of which, about nine percent (4903 PLHIV) died during the project period, i.e., between 2006–2011. Table 1 shows the basic socio-demographic and health-related selected characteristics of PLHIV reveals that, at the overall level, of the total PLHIV, majority of them were aged 18 and above (91 percent) with a mean age of 34 years, 52 percent were female, about 84 percent were ever-married, more than half of them were literate (53 percent), about two third were involved in income generation activities, and around 35 percent from urban areas. On the clinical part, at the overall level, half of the PLHIV were on ART, about one-third had the CD4 count 350 and above, and about 14 percent diagnosed with TB during the study period. While 46 percent PLHIV were recently exposed to the intervention (about six months ago), one-fourth of the PLHIV were exposed to the intervention for 24 or more months. The median duration since HIV diagnosis was found to be 17 months in general.

**Table 1:** Differential in the profile of PLHIV registered with the programme by their survival status.

	Total	Alive	Died	p-value*
**N**	**55,801**	**50,898**	**4,903**	-
**A. Background characteristics**				
% aged 18+ years	90.6	90.2	94.8	<0.001
Mean age (SD)	34.1 (11.9)	33.9 (11.9)	35.9 (11.3)	<0.001
% female	51.5	52.6	39.5	<0.001
% ever married	83.5	83.2	86.5	<0.001
% literate	52.6	53.2	46.5	<0.001
% working/employed	66.4	66.2	68.8	<0.001
% belong to urban areas	35.3	35.9	28.8	<0.001
**B. Clinical Characteristics**				
% on ART	50.3	51.5	37.9	<0.001
% with CD4 count not done	24.5	22.9	41.9	<0.001
% with CD4 count <100	9.5	8.3	21.9	<0.001
% with CD4 count 100–249	18.8	18.8	18.9	0.967
% with CD4 count 250–349	13.5	14.2	6.8	<0.001
% with CD4 count 350+	33.6	35.8	10.5	<0.001
% had TB at any stage^#^	14.0	12.9	24.9	<0.001
**C. Duration of programme exposure**				
% exposed for 1–6 months	46.0	43.4	73.1	<0.001
% exposed for 7–11 months	8.8	8.8	9.6	0.039
% exposed for 1–2 Years	20.0	20.8	12.1	<0.001
% exposed for more than 2 years	25.1	27.0	5.1	<0.001
Median duration since HIV diagnosis (in months)	17	18	11	

^#^ever had TB since tested positive and last contact prior to end of intervention.

*differences of two-proportions were performed using z-test.

The analysis presented in Table 1 also depicted that the PLHIV who remained alive during the study period or who died in between, significantly differed from each other in terms of their socio-demographic, clinical and programme exposure related characteristics. For instance, the higher proportion of PLHIV those remained alive were female, literate, belonging to urban areas, were on ART, and had CD4 count above 350. Moreover, a larger proportion of surviving PLHIV had a longer duration of programme exposure. On the contrary, the proportion of PLHIV, who died during the interim intervention period, majority of them were aged 18 and above, ever married, were involved in income generation activities, did not undergo counting of CD4 and suffered from TB. A significantly higher proportion of PLHIV who died had less than six months of programme exposure.

### Estimation of mortality rates

**Fig 1:**
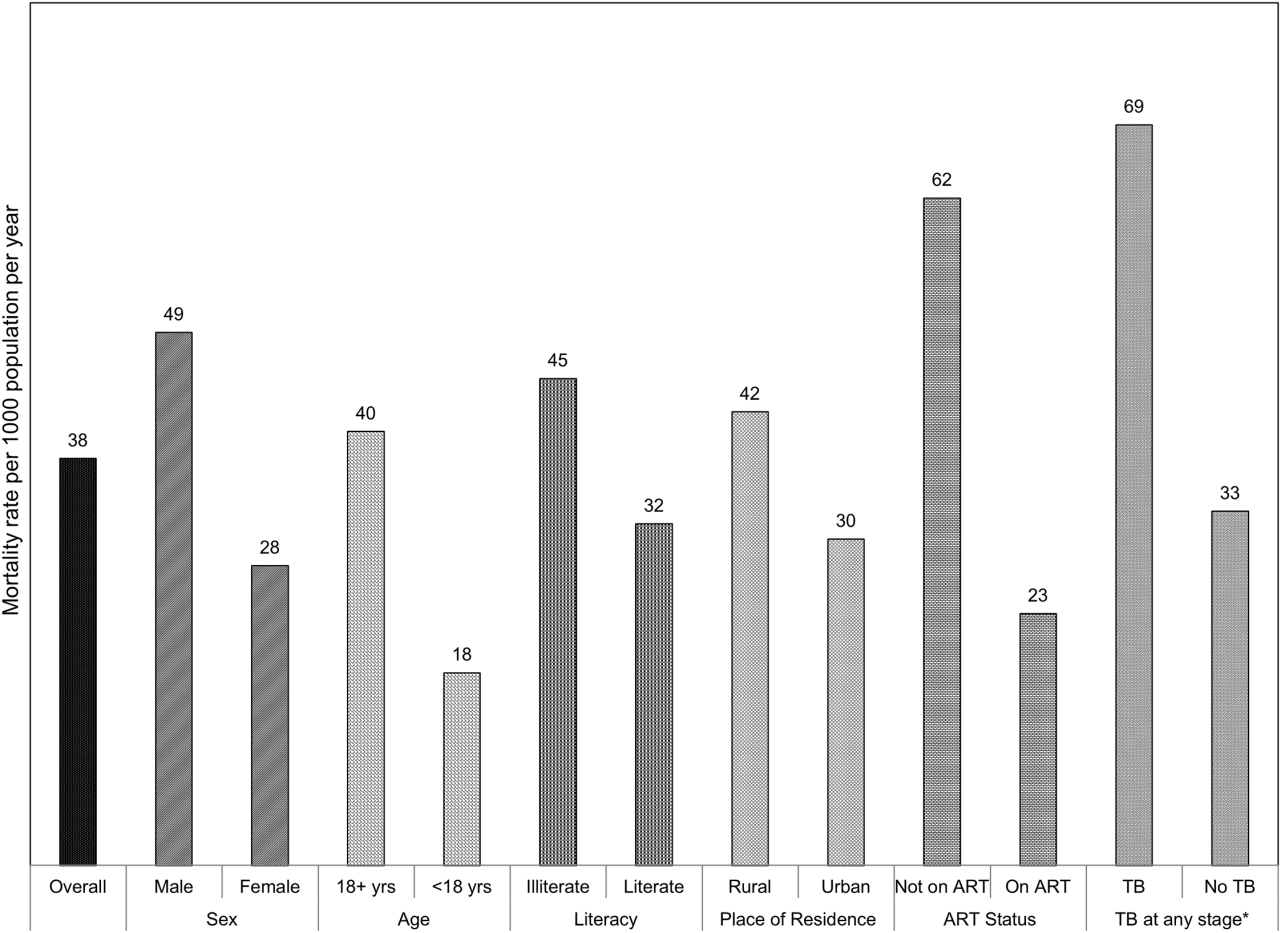
Mortality rates among PLHIV by selected background characteristics. The bar diagram demonstrates the estimated mortality rates among PLHIV per 1000 population per year. The estimated mortality rates are also disagreegated by sex of the PLHIV, their age, literacy, place of residence, ART status and presence of co-infection like TB at any stage. TB at any stage is defined as ever had TB since tested positive and last contact prior to end of intervention.

Fig 1 shows the mortality rates among PLHIV by their characteristics. While the overall death rate was 38 deaths per 1000 persons per year, PLHIV, who were male, aged 18 and above, illiterate, and belonging to rural areas had higher mortality rates compared to their respective counterparts. On the other hand, higher chances of survival were noticed among those on ART and those without co-morbidity such as TB, compared to their respective counterparts.

### Kaplan-Meier survival probabilities

**Fig 2:**
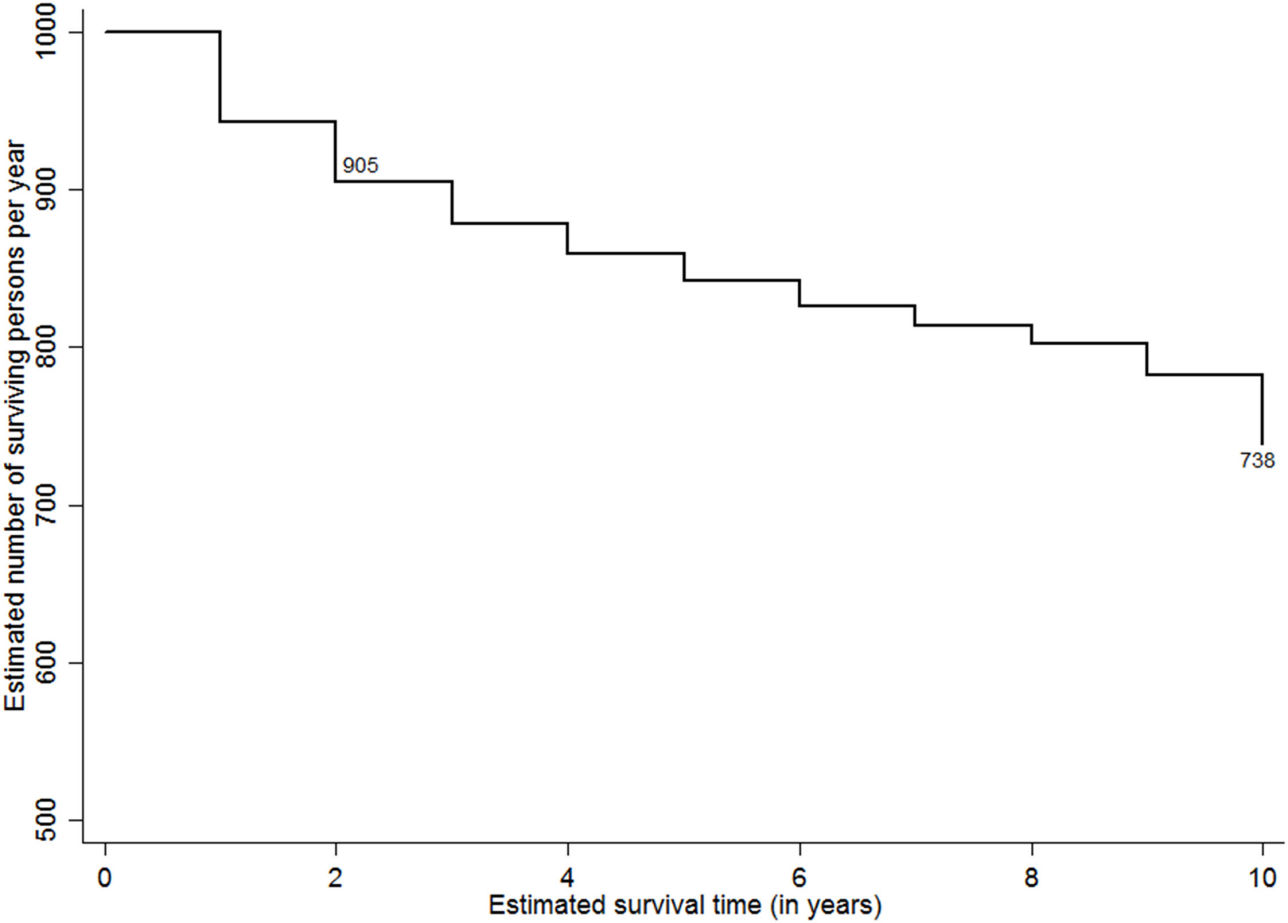
Adjusted Kaplan-Meier survival estimates (per 1000 persons per year). The line graph shows ten-year survival probability curves among PLHIV per 1000 persons per year since they tested positive for HIV at the overall level. The estimates are adjusted by the place of residence.

**Fig 3:**
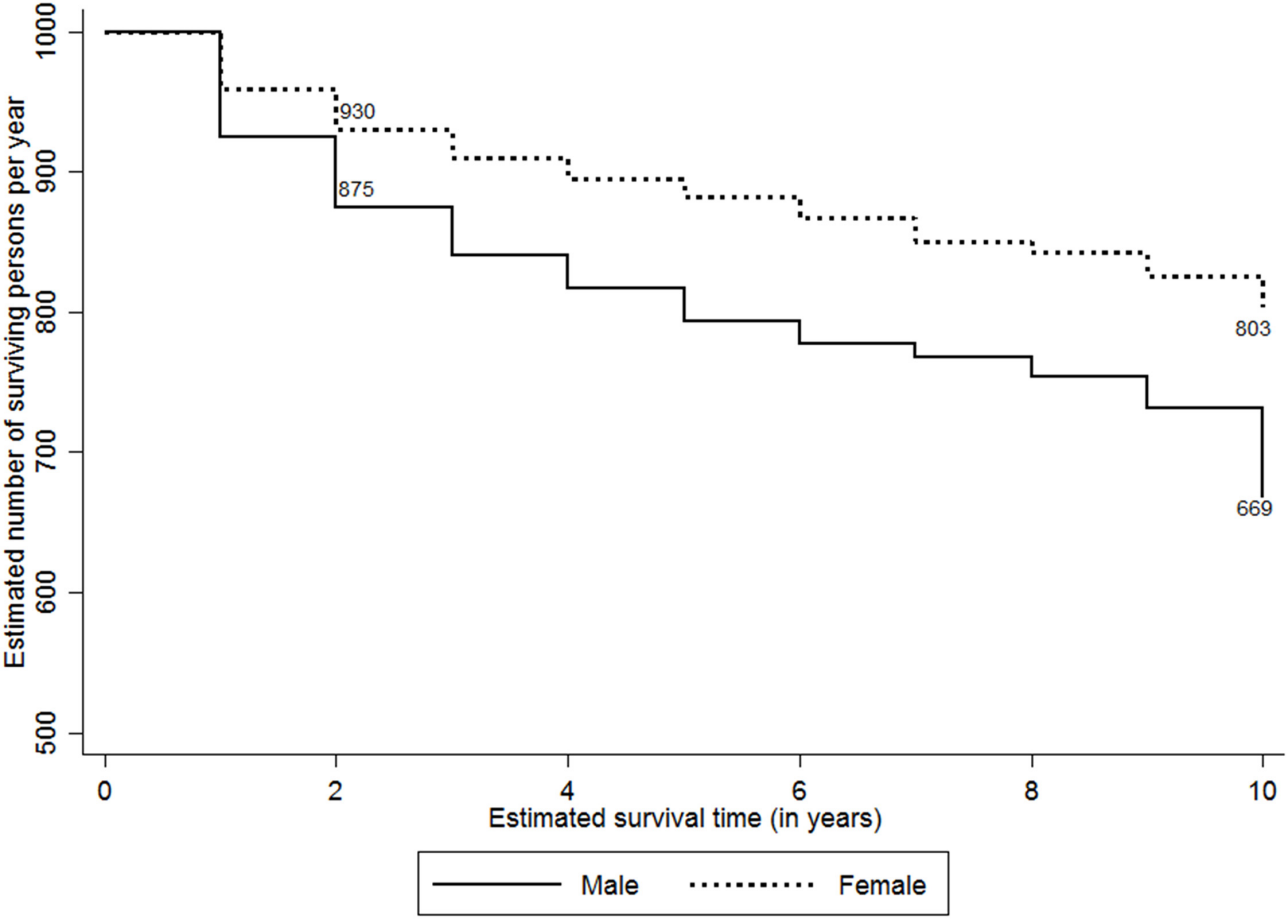
Adjusted Kaplan-Meier survival estimates by sex (per 1000 persons per year). The line graph shows ten-year survival probability curves among PLHIV per 1000 persons per year since they tested positive for HIV by sex of the PLHIV. The estimates are adjusted by their place of residence.

**Fig 4:**
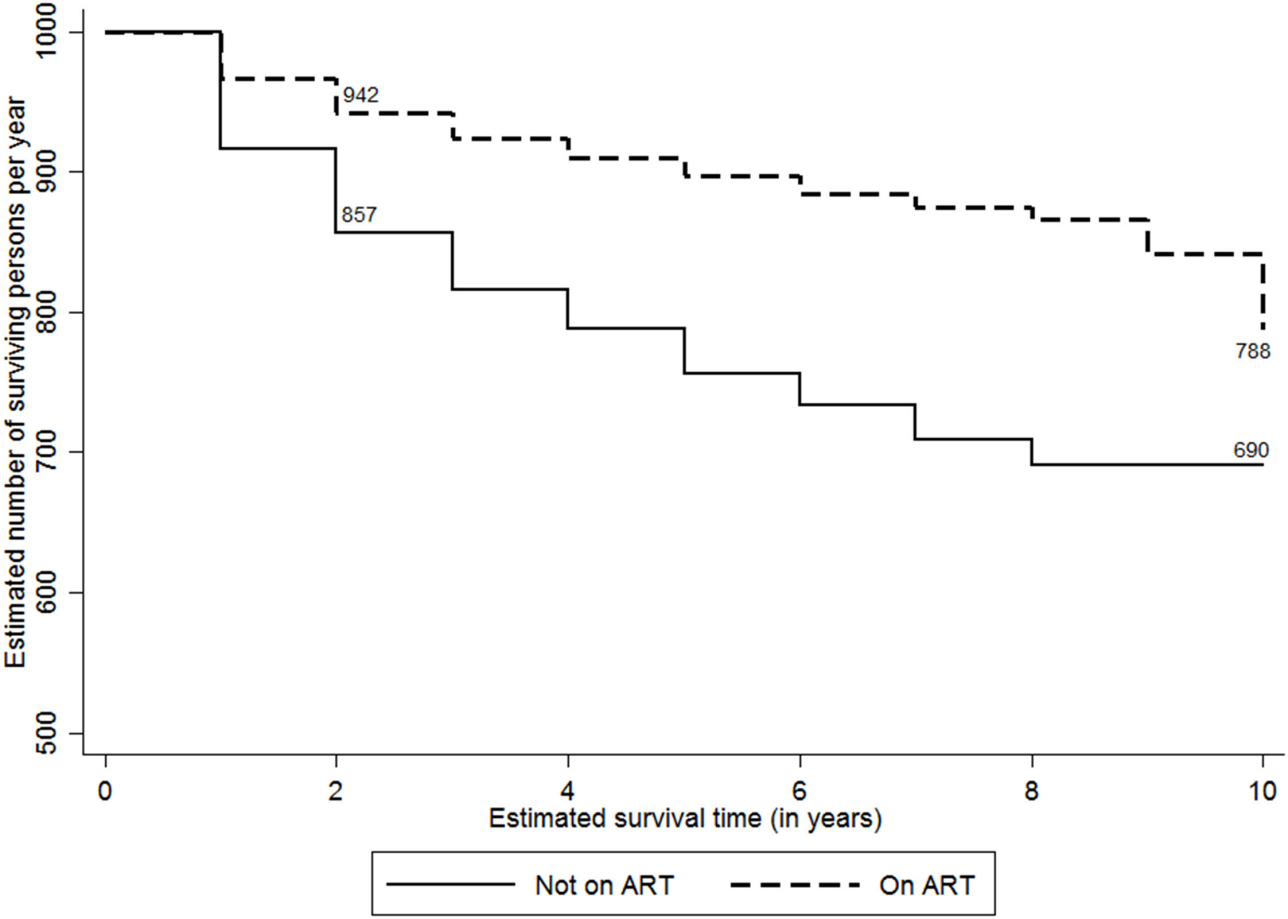
Adjusted Kaplan-Meier survival estimates by ART status (per 1000 persons per year). The line graph shows ten-year survival probability curves among PLHIV per 1000 persons per year since they tested positive for HIV by their ART status. The estimates are adjusted by the place of residence of PLHIV.

**Fig 5:**
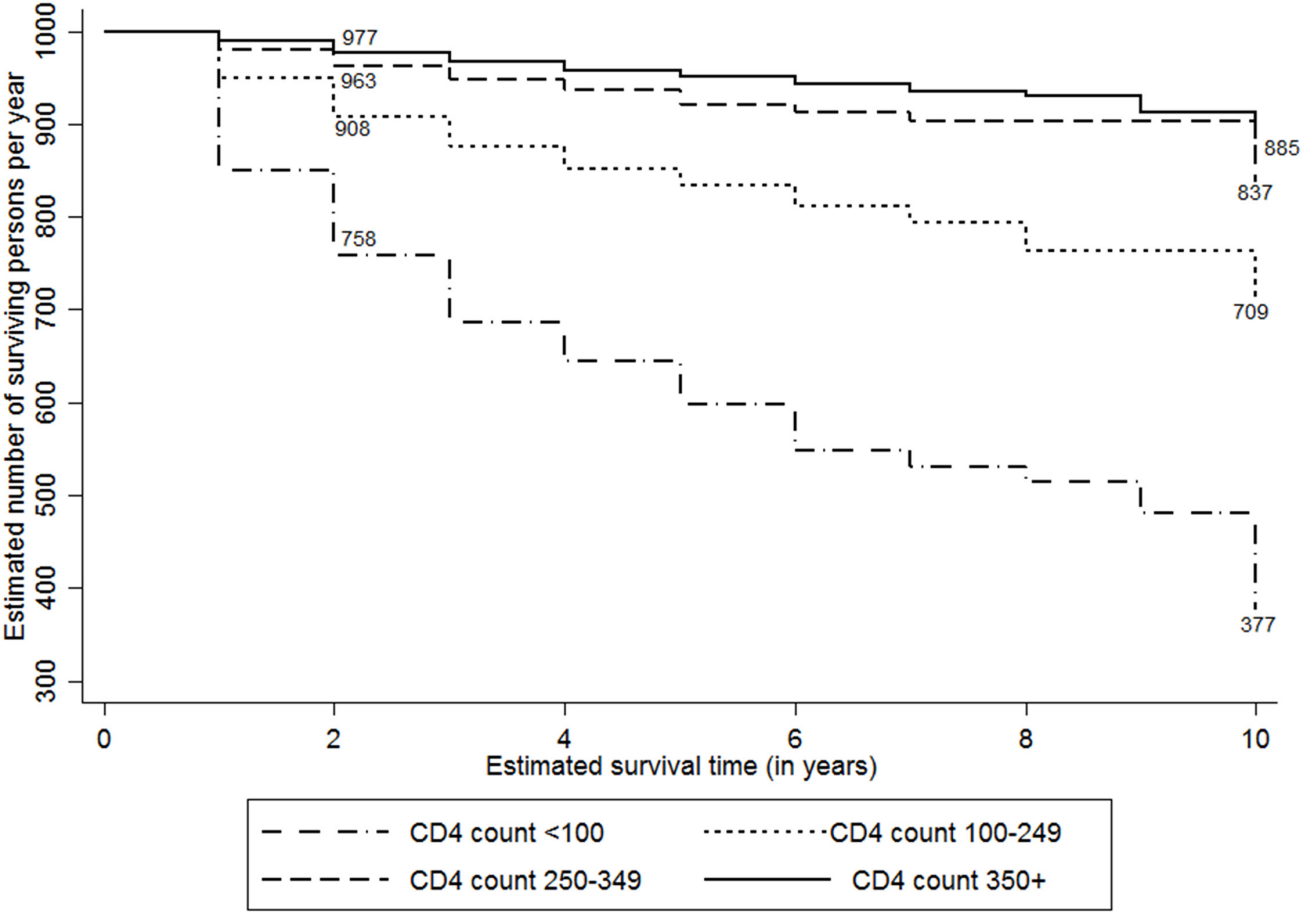
Adjusted Kaplan-Meier survival estimates by CD4 count (per 1000 persons per year). The line graph shows ten-year survival probability curves among PLHIV per 1000 persons per year since they tested positive for HIV by CD4 status. The estimates are adjusted by their place of residence. The CD4 staus is divided into four categories, i.e.CD4 count less than 100, CD4 count 100–249, CD4 count 250–349 and CD4 count 350 or more.

The study attempted to analyse the expected survival probability of PLHIV post they detected positive by selected characteristics such as sex, ART and CD4 status, and their duration of programme exposure. Considering the variations in accessibility to HIV care services by their place of residence (i.e., rural and urban), the survival probabilities were adjusted for their location of usual stay. Fig 2 shows the ten-year adusted survival probability curves among PLHIV since they tested positive for HIV. At the overall level, 738 PLHIV per 1000 person years expected to survive post ten years since they tested positive. The adjusted survival rates were higher among females (803 per 1000 person years) compared to males (669 per 1000 person-years) (Fig 3). Figs 4 and 5 show the ten-year adjusted survival probability curves for PLHIV by ART and CD4 status, respectively. As expected, those who were on ART had a higher likelihood of survival (788 per 1000) compared to those not on ART (690 per 1000) post ten years since tested HIV-positive. Similarly, increase in CD4 count from less than 100 to 249 increased the survival rate from 377 to 709 per 1000 person years, post ten years since tested HIV-positive. A further increase in CD4 count increased the survival rate to 885 per 1000 for the PLHIV having CD4 count 350 and above.

**Fig 6:**
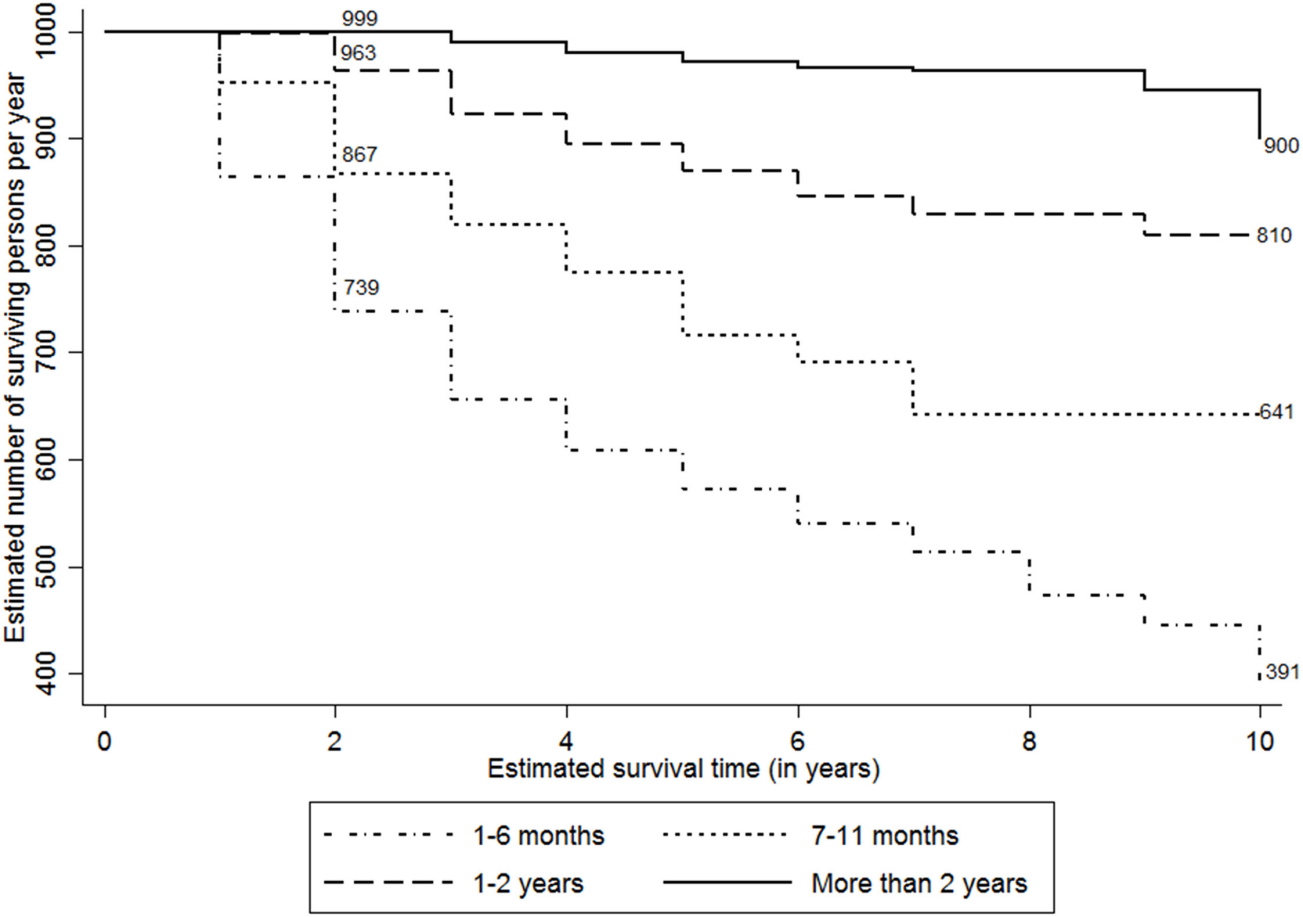
Adjusted Kaplan-Meier survival estimates by duration of programme exposure (per 1000 persons per year). The line graph presents the adjusted survival probability curves among PLHIV per 1000 persons per year since they tested positive for HIV by duration of programme exposure. The estimates are adjusted by the place of residence of PLHIV. The duration of exposure to programme is divided into four categories, i.e. 1 to 6 months, 7 to 11 months, 1–2 years and more than 2 years.

Fig 6 presents the adjusted survival probability curves for the duration of programme exposure for PLHIV post ten year period since they tested HIV-positive. As expected, there has been a significant increase in the survival rates with a gradual rise in the duration of programme exposure. For instance, the ten-year survival rates post detection to HIV increased from 391 per 1000 to 900 per 1000 person-years among those who had at 2 or more years of programme exposure compared to the PLHIV with less than six months of programme exposure, respectively.

The effect of programme exposure, ART treatment, and CD4 counts may be confounded by the background characteristics of PLHIV, and hence, survival probabilities can be tentative until they are adjusted for the effects of background factors. Table 2 presents the results of Cox Proportional Hazard Model estimating the risk of mortality by the associated risk factors. The analysis confirmed the already described findings that duration of programme exposure, being on ART, and higher CD4 counts reduced the mortality risks among PLHIV even after adjusting for potential confounding effects of the selected factors considered in the study. For instance, the mortality risk among PLHIV increases significantly with the increase in age (HR: 1.461; CI: 1.241–1.721) and due to presence of TB as co-infection (HR: 1.796; CI: 1.674–1.926) but decreases among females (HR: 0.679; CI: 0.636–0.725), those literate (HR: 0.690; CI: 0.650–0.733), working (HR: 0.848; CI: 0.791–0.908), and belong to the urban areas (HR: 0.706; CI: 0.662–0.753). Furthermore, being on ART and increase in CD4 count from less than 100 to 100–249 significantly reduces the mortality risk among PLHIV (HR: 0.627; CI: 0.585–0.673 & HR: 0.618; CI: 0.566–0.676, respectively). A further increase in CD4 count from 250 to 349 and from 350 and above reduces the death rate significantly. All these relationships were found to be statistically significant at 1 percent level of significance.

**Table 2:** Results from Cox proportional hazard model depicting the risk of mortality by selected socio-demographic, clinical and programme exposure related characteristics.

	Adjusted* hazard ratio	[95% CI]	P>|z|
**Age**			
< 18 years^®^			
18+ years	1.461	(1.241–1.721)	<0.001
**Gender**			
Male^®^			
Female	0.679	(0.636–0.725)	<0.001
**Marital status**			
Never Married^®^			
Ever Married	1.088	(0.980–1.208)	0.114
**Literacy status**			
Illiterate^®^			
Literate	0.690	(0.650–0.733)	<0.001
**Work status**			
Not working/unemployed^®^			
Working/employed	0.848	(0.791–0.908)	<0.001
**Place of residence**			
Rural^®^			
Urban	0.706	(0.662–0.753)	<0.001
**ART status**			
Not on ART^®^			
On ART	0.627	(0.585–0.673)	<0.001
**Most recent CD4 count result**			
CD4 count not done^®^			
CD4 count <100	1.186	(1.088–1.293)	<0.001
CD4 count 100–249	0.618	(0.566–0.676)	<0.001
CD4 count 250–349	0.355	(0.315–0.401)	<0.001
CD4 count 350+	0.234	(0.211–0.260)	<0.001
**TB at any stage**^#^			
No TB^®^			
TB	1.796	(1.674–1.926)	<0.001
**Duration of programme exposure**			
1–6 months^®^			
7–11 months	0.649	(0.589–0.716)	<0.001
1–2 Years	0.254	(0.232–0.279)	<0.001
More than 2 years	0.062	(0.054–0.071)	<0.001

*Adjusted for age, sex, marital status, education attainment, occupation, place of residence, ART status, most recent CD4 count result, TB status and duration of programme exposure.

^#^Ever had TB since tested positive and last contact prior to end of intervention.

^®^ Reference category.

The most important result to notice from Table 2 is that PLHIV having at least one year of exposure to the intervention (1–2 years) had significantly lower risk of mortality (HR: 0.254; CI: 0.232–0.279); which subsequently reduced to 0.062 (CI 0.054–0.071) among those who had more than two years of intervention exposure while comparing with the PLHIV exposed to the intervention for just 6 months.

## Discussion

Based on the data derived from a retrospective cohort of patients living with HIV and registered with an intensive HIV care and support programme for PLHIV in selected districts of Karnataka, this study made an attempt to estimate the mortality rate and the probability of survival post ten-year intervention period among them. Only a few studies in India, till date, have made any effort to determine the mortality risk using the programme-based individual tracking data using a large sample size. Findings of this study documented the death rate of 38 per 1000 person-years, which was little higher than the death rates reported in the other parts of the country.[10, 12]

The study reported relatively higher mortality rates among males compared to females. This finding is somewhat contrary to the general belief that men have better access and utilization of health care services including HIV-testing and counseling, thereby, should have lower chances of mortality. However, because the intervention was primarily focussed on rural areas, the social stigma attached around HIV/AIDS may lead to the lower access of services among men. The role of stigma in limiting the access of services may not be so strong among females as the intervention had several focused activities to bring the females to the clinic periodically and serve them various health services that they need.[8] Existing studies have well documented the prevailing high levels of stigma associated with the HIV, at the community as well as the family level, in the similar type of rural context.[15] A higher number of male deaths as compared to females have been noticed in other studies also.[10, 12, 16–18] Such deaths are mostly contributed to a higher rate of loss to follow-up or delay in the initiation of ART at the most advanced stage of AIDS among males compared to females.[10] Programmatically, no doubt it is important to make females aware of the symptoms of HIV, places where HIV testing facility is available, as well as early detection of the infection, but more importantly males should also be intensively covered with the intervention so that they may enroll themselves in the care and support programme if detected positive. The higher deaths among males have also attributed to the biological differences in the drug response to ART among males and females.[10, 18]

Both bivariate and multivariate analysis showed a strong association between higher mortality rates and demographic variables such as age, educational level, and place of residence. The higher death rate among older ages has been found in other studies too.[10, 12, 19–25] Findings in these studies clearly indicated that the risk of mortality significantly increases with the increase in age while decreases with the increase in educational attainment.[12] The inadequate medical facility in the rural areas is found to be a contributing factor for the higher risk of mortality among HIV-patients in rural areas.[20–22, 24]

In agreement with the previous studies, the findings of this study also suggest lower death rate and a higher likelihood of survival among the PLHIV, who were on ART. Globally, in the absence of a cure for HIV, the increased use of ART has been able to improve the survival of individuals infected with HIV[26–28] by postponing their progression to death.[29, 30] The introduction of ART resulted in significant decline in the AIDS-related deaths not only in developed countries but also in India.[31–35] According to NACO, as a result of the extensive availability of ART therapy and increased uptake of these services has led to fewer AIDS-related deaths in India during 2011 than a decade ago.[2, 31] As a part of the initial assessment on the effectiveness of ART programme in India, a study from southern India reported a decline in the death rate from 25 deaths per 100 person years to 5 deaths among young PLHIV soon after initiation of ART.[36]

One of the significant finding of our study is that the likelihood of survival increases with the high level of CD4 count and it corraborates with the previous studies conducted in India.[37, 38] The chances of survival found in these studies were comparatively lower among the HIV-positive patients having lower CD4 count. Findings of our study clearly demonstrated the cascade of mortality among the PLHIV with the declining CD4 count. The minimum likelihood of death was observed among the PLHIV having CD4 count 350 and above whereas it was highest among those having the CD4 count less than 100. Previous studies have also found a relationship between low CD4 count and presence of opportunistic infection. For instance, PLHIV having CD4 count less than 200 at the time of HIV diagnosis had higher chances of acquiring other opportunistic infections such as TB.[31] Findings of our study also suggested the increased risk of mortality among the PLHIV diagnosed with other co-morbidities such as TB. Tuberculosis has appeared as one of the leading causes of deaths among patients infected with HIV.[31, 32] A higher proportion of deaths observed among HIV-positive individuals suffering from multiple infections, especially TB.[39, 40] Studies in India have clearly documented that PLHIV acquired selected opportunistic infections such as TB, oral candidiasis, and few other non-communicable diseases were at much higher risk of mortality compared to their counterparts having no such infections.[41–44]

Analysis conducted in this paper also focused on analyzing the effect of exposure to care and support intervention on the survival of PLHIV. It is clear that the survival probabilities improved as the duration of programme exposure increased. However, if the programme exposure is more than two years, there is hardly any further decrease in the survival probability post ten years since tested positive. Such relationship, of course, expected to depend upon CD4 count and whether the person is on ART or not. But results of the hazard model indicate that even after controlling for CD4 count and being on ART along with other background characteristics, the programme exposure alone reduces the risk of mortality drastically compared to who did not have any intervention exposure. The intervention exposure should have helped PLHIV to undergo the CD4 count test regularly and to get on ART treatment whenever necessary. However, intensive programme exposure for two years appears to be sufficient to sustain this behaviour.

Despite, the study observed various important findings including the minimum duration of intervention exposure required to reduce the mortality risk among PLHIV, the findings should be considered with caution. As the study was taken up using the retrospective data, some of the potential confounders of mortality such as viral load, previous histories of ART uptake, sequencing of co-infection, and side effects during the ART regime under the process of medication could not be included. The information on opportunistic infections, other than TB, was not properly captured. Similarly, the information on distance to ART center was not available. Therefore, our analysis could not use any information pertaining to the previous history of ART uptake presence of other opportunistic infections. Our results on the death rate might be overestimated due to the fact that all causes of death were included in the study and it is possible that certain deaths might have occurred due to non-HIV related causes. Due to a very large sample size, most of the bi-variate and multivarite results are statistically significant at one percent level of significance, and therefore, results should again be considered with caution. However, despite these limitations, many of the findings of this paper have importance from the planning of care and support programme and its implementation.

## Conclusions

The study revealed several useful results that have potential bearing on reducing the risk of mortality among the HIV-infected population. The most useful findings emerged from our analysis are adjusted hazard risks for CD4 status and duration of programme exposure. The increase in CD4 count from less than 100 to more than 350 reduced the hazard risk by more than 90 percent. An important question that was not properly answered till date was the required level of the CD4 count to reduce the hazard risk to zero. In fact, the even more relevant question to be asked is: what is an optimum level of the CD4 count for ART treatment? Our findings clearly demonstrated that a minimum CD4 count of 100 is required for the survival, whereas, CD4 count of more than 350 enhances the survival chances of PLHIV by multiple times. To maintain a high level of CD4 cell count among PLHIV, it is essential for the national programme to focus on the adherence of HIV-infected persons to ART therapy. Though the efforts are being made to improve the ART adherence among HIV-infected people and provision of free ART medicines at designated government ART centres have already been made under the national programme, still regular follow-up with the HIV-positive patients is required for better results. Further research is required to understand the reasons for non-adherence. Addressing the adherence in the ongoing ART programme in India may bring a notable reduction in the current level of mortality among HIV-infected persons.

Another significant finding of our paper is that the programme exposure of at least two years can brings a substantial reduction in the likelihood of mortality among PLHIV. More specifically, a focused care and support intervention with repeated follow-up and strategic use of information for ongoing improvement in the health and well-being of PLHIV has been critical in reducing the mortality. The project like Samastha that focused on medical and psycho-social services, regular follow-up of CD4 counts, and ART adherence contributed significantly to reduce mortality among PLHIV. For policy makers and programme implementers, it is definitely encouraging to know that exposure to a well-designed programme can become a good proxy for monitoring of CD4 counts and ART adherence.
